# Modified Versus Conventional Prone Position for COVID‐19 Patients in Adult Intensive Care Units: A Comparative Study

**DOI:** 10.1111/nicc.70122

**Published:** 2025-07-27

**Authors:** Chen Huang, Wenwen Qi, Shuyuan Zhao, Yan Jiang, Yirong Sun, Xuelian Xu, Chaowei Yao, Xiaoye Wang, Enqiang Mao, Feng Jing, Erzhen Chen

**Affiliations:** ^1^ Nursing Department Ruijin Hospital, Shanghai Jiaotong University School of Medicine Shanghai China; ^2^ Department of Emergency Ruijin Hospital, Shanghai Jiaotong University School of Medicine Shanghai China; ^3^ The Dean's Office, Ruijin Hospital, Shanghai Jiaotong University School of Medicine Shanghai China

**Keywords:** COVID‐19, pressure ulcer, prone position, respiration artificial, retrospective comparative study

## Abstract

**Background:**

Prone position has been reported to improve prognosis and reduce mortality in COVID‐19 patients, but poor patient tolerance and complications remain an issue.

**Aim:**

This study aimed to compare the modified vs. conventional prone position on pressure injuries in COVID‐19 patients in adult intensive care units (ICU).

**Study Design:**

This retrospective comparative study enrolled COVID‐19‐positive patients who were admitted to the emergency adult ICU of a tertiary general hospital between December 2022 and January 2023. All data were extracted from patient charts. The primary outcome was pressure injury. During the study period, pressure injuries were evaluated using the staging system of the International Clinical Practice Guideline for the prevention and treatment of pressure injuries in 2019.

**Results:**

A total of 39 COVID‐19‐positive patients (16 females) were included, and 19 patients received the modified prone position. Compared to those with the conventional prone position, patients with the modified prone position had significantly lower occurrence of pressure injury (3 (25.0%) vs. 9 (75.0%), *p* = 0.044), eyelid oedema (2 (10.5%) vs. 9 (45.0%), *p* = 0.031) and facial oedema (5 (26.3%) vs. 13 (65%), *p* = 0.024) and significantly higher daily continuous prone position time (9.16 ± 3.01 vs. 6.50 ± 2.14, *p* = 0.003). The occurrence site and stage of pressure injury, transcutaneous blood oxygen saturation, airway adverse events and brachial plexus injury were comparable between the groups (all *p* > 0.05).

**Conclusion:**

Compared to the conventional prone position, the modified prone position may significantly reduce the occurrence of pressure injuries and improve patient tolerance in COVID‐19 patients in the adult ICU.

**Relevance to Clinical Practice:**

These findings provide guidance for critical care nurses to implement prone positioning interventions.

**Summary**

- What is known about the topic
  ○ Prone ventilation can improve the oxygenation of patients with COVID‐19; however, patients in the conventional prone position had poor tolerance to ventilation, resulting in a high incidence of unplanned extubation, pressure injuries and difficulty in nursing.
- What this paper adds
  ○ The difference between the modified and conventional prone position positions is the patient's head, where legs are usually placed, and the use of a U‐shaped pillow in the modified prone position.
  ○ Compared to the conventional prone position ventilation, modified prone position ventilation has significantly lower occurrence rates of pressure injury, eyelid oedema, facial oedema and significantly higher daily continuous prone position time.

## 1 Introduction

Prone position has been widely used as a lung protective ventilation strategy in clinical settings [1, 2, 3]. A previous study revealed that prone ventilation can improve the oxygenation of patients with acute respiratory distress syndrome (acute respiration dysfunction syndrome, ARDS) [4]. In another study, Weiss et al. reported that the conventional prone position is widely used in the treatment of patients with moderate to severe ARDS and severe pneumonia, including severe or critical COVID‐19 patients [5]. *China's Diagnosis and Treatment Plan for Novel Coronavirus Pneumonia* pointed out that COVID‐19 patients with severe high‐risk factors and rapid disease progress should be treated in a standard prone position, and it is recommended that this position be maintained for no < 12 h a day [6].

According to a research report of the clinical treatment expert group of novel coronavirus pneumonia in Shanghai, the treatment of COVID‐19 patients with hypoxaemia in the prone position can effectively increase oxygenation and decrease intubation rate, thus improving prognosis and reducing mortality [7]. However, patients in the prone position had poor tolerance to ventilation, resulting in a high incidence of airway adverse events, pressure injuries and difficulty in nursing [8, 9].

Luccini et al. showed that complications during prone position treatment were mainly related to the time duration spent in one single prone position and the overall time spent on multiple cycles of the prone position, which could be affected by the expertise of the medical team and nursing quality. The incidence of complications varies in reports, but appropriate management can reduce the incidence of complications to 1% [10].

To effectively decrease the incidence of complications in the clinical prone position treatment and improve patient tolerance, we propose the improved posture in the prone position based on data obtained by a micro‐group focus interview on the conventional prone position. The focus group was composed of five clinical nursing experts, including two first‐line clinical nurses, two head nurses in the emergency care room and one expert in hospital nursing management. Their average age was 40.2 years, with an average work experience of 19.4 years. The modified prone position places the patient's head where the legs were usually placed. This position is designed to elevate the patient's chest and keep the head in a relatively horizontal position. A U‐shaped pillow is utilised instead of the traditional cushion for the head to provide ample breathing room and accommodate catheters for oxygen therapy and respiratory support through an opening. This study aimed to explore the effect of the modified prone position in COVID‐19 patients in intensive care units (ICUs).

## 2 Aims and Objectives of Study

Description of the modified prone position and verification of its effectiveness.

## 3 Design and Methods

This study was reported following the STROBE statement and checklist [11].

### 3.1 Setting and Sample

This retrospective comparative study enrolled COVID‐19‐positive patients who were admitted to the emergency ICU of a tertiary general hospital in Shanghai, China, between December 2022 and January 2023. The unit has 19 beds and admits critically ill patients, with more than 400 critically ill patients admitted annually.

Inclusion criteria were as follows: (1) patients diagnosed with COVID‐19 infection according to the Diagnosis and Treatment Plan for novel coronavirus Pneumonia (Trial Version 9) for COVID‐19 infection [6]; (2) age ≥ 18 years old; (3) patients who received non‐invasive mechanical ventilation without endotracheal intubation due to severe hypoxaemia; and (4) patients undergoing prone position treatment. Exclusion criteria were as follows: (1) a risk of airway obstruction: the doctors evaluate that patients have symptoms such as shortness of breath, difficulty breathing, chest tightness and cough. In severe cases, it may lead to purple lips, shock and other conditions. Combining chest X‐ray examination and bronchoscopy examination for judgement; (2) respiratory failure caused by cardiogenic pulmonary oedema; (3) SpO_2_ cannot be accurately tested or continuously monitored; (4) unstable haemodynamics, and vasoactive drugs are needed (under the premise of full‐volume resuscitation and systolic blood pressure < 90 mmHg or mean arterial pressure < 65 mmHg); (5) glaucoma or other sharp increase in intraocular pressure; intracranial hypertension caused by craniocerebral injury; (6) a high risk of pulmonary embolism according to the consensus of Chinese experts on the diagnosis and treatment of acute pulmonary embolism (2023); strong risk factors include major trauma, surgery, lower limb fractures, joint replacement and spinal cord injury.

### 3.2 Data Collection Tools and Methods

In this retrospective study, all data were extracted from the patient charts using the laboratory and examination reports and the nurses' notes. All data found in the charts were recorded by healthcare professionals possessing certifications appropriate for their functions. The patients were evaluated by the attending physicians, who all belong to the same group and follow the same guidelines and standards. Data extraction completeness and accuracy were ensured through a stringent data collection protocol, which included electronic medical record verification and cross‐checking by two independent researchers. Demographic data and clinical characteristics were recorded, including sex, gender, body mass index (BMI), Charlson Comorbidity Index, Acute Physiology and Chronic Health Evaluation (APACHE) II score at the beginning of the study, PaO_2_, PaCO_2_, sequential organ failure assessment (SOFA) score at the beginning of the study, time from onset of symptoms to admission, time from admission to the first prone position treatment, C‐reactive protein, D‐Dimer, lactic acid, pressure injury, airway adverse events, eyelid and facial oedema, brachial plexus injury, the tolerance of prone position treatment, ventilation mode and transcutaneous blood oxygen saturation before and after treatment. Percutaneous blood oxygen saturation was measured by non‐invasive pulse oxygen saturation.

APACHE II scoring system provides an excellent method for classifying the severity of critically ill patients with increased sensitivity and specificity (sensitivity = 28% and specificity = 99.5%) [12]. The staging of pressure injuries was conducted according to the latest definition and staging system of the International Clinical Practice Guideline for the prevention and treatment of pressure injuries in 2019 [13].

The stages are defined as follows:

Stage I: Non‐blanchable erythema of intact skin.

Stage II: Partial‐thickness skin loss with exposed dermis.

Stage III: Full‐thickness skin loss.

Stage IV: Full‐thickness skin and tissue loss.

Unstageable: Full‐thickness skin and tissue loss with the extent of damage obscured.

Deep Tissue Injury (DTI): Persistent non‐blanchable deep red, maroon or purple discolouration.

Brachial plexus injury refers to a kind of peripheral nerve injury caused by various reasons, after which the upper limb function of the patient is partially or completely lost. The tolerance of prone position treatment was evaluated by comparing the duration of prone position in two groups of patients with non‐invasive mechanical ventilation.

Head Oedema Assessment: The assessment employed a self‐developed head oedema‐specific assessment scale, which was based on McGee's four‐grade classification of pitting oedema [14] and the WHO's definition of pathological oedema [15], with adaptive modifications considering the anatomical characteristics of ICU patients. Clinical nurses conducted the assessment based on the following criteria. Grade 0 (no oedema): No swelling of the eyelids or surrounding skin and no indentation upon palpation. Grade 1 (mild oedema): Slight swelling of the eyelids, with an indentation that recovers quickly (< 2 s) after pressure. Grade 2 (moderate oedema): Noticeable swelling of the eyelids, with an indentation that takes longer to recover (2–5 s) after pressure. Grade 3 (severe oedema): Significant eyelid swelling, with an indentation that persists (> 5 s) after pressure, possibly accompanied by skin shininess or discoloration.

Airway adverse events: Airway events specifically included artificial airway obstruction and artificial airway dislodgement, as assessed by nurses.

Brachial plexus injury [16]: Muscle strength grading was commonly used to evaluate the function of muscle groups innervated by the affected nerves. The system used during the study period was the Medical Research Council (MRC) muscle strength grading scale, which classifies muscle strength into six levels (Grades 0–5). Grade 0: Complete paralysis, no muscle contraction. Grade 1: Slight muscle contraction but no joint movement. Grade 2: Joint movement possible only after eliminating gravity (e.g., on a horizontal plane). Grade 3: Full range of joint movement against gravity but without resistance. Grade 4: Joint movement possible against resistance, but muscle strength is weakened. Grade 5: Normal muscle strength, full movement against strong resistance.

#### 3.2.1 Modified Prone Position

**FIGURE 1:**
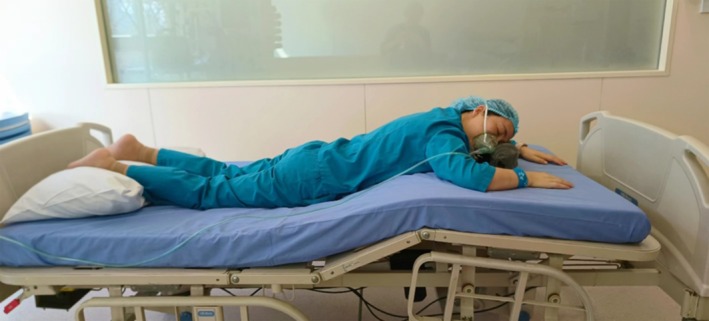
Modified prone position. Adapted with permission from Department of Emergency, Ruijin Hospital, Shanghai Jiaotong University School of Medicine, Shanghai, China.

An improved prone position was applied in patients. The head and foot of the bed were turned in opposite directions, positioning the patient's head where the legs were usually placed. When the head side of the bed was raised, the chest could be elevated while the head was kept in a relatively horizontal position. The patient's arms were placed alongside the body, slightly bent at the elbows, with hands placed at the level of the head. The patient's head was placed on a U‐shaped pillow, and the lower limbs were positioned on cushions to ensure a comfortable position (Figure 1).

The steps to achieve the modified prone position were as follows: (A) the patient was transferred to the treatment bed; (B) the head of the patient's bed was turned to align it with the patient's head; (C) the pad was placed as required; (D) the patient was guided/assisted during the transfer from the treatment bed to the hospital bed; the patient was in the prone position, with the head at the foot of the bed, leaning to one side; and (E) the position of the pillow was properly adjusted to protect bone protrusions, and the height of the foot of the bed was adjusted to ensure the comfort of the patient. All patient transfers were performed using sliding sheets.

#### 3.2.2 Conventional Prone Position

**FIGURE 2:**
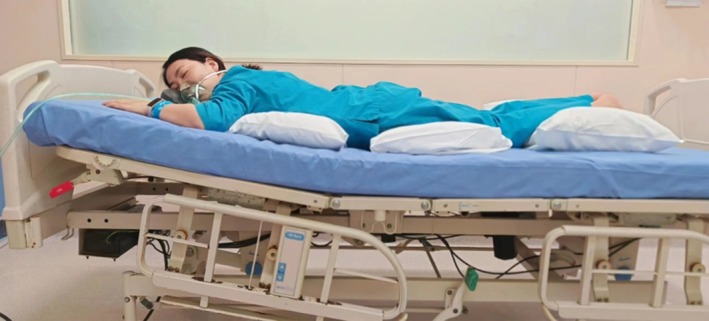
Conventional prone position. Adapted with permission from Department of Emergency, Ruijin Hospital, Shanghai Jiaotong University School of Medicine, Shanghai, China.

The patient was in the prone position, with his head turned to one side or supported on a U‐shaped pillow, and several soft cushions were placed under the patient's body to ensure a comfortable prone position. The head of the bed was raised by about 10°, and the patient's hands were in a freestyle position (Figure 2).

### 3.3 Data Analysis

No missing data were encountered in this study. Statistical analyses were performed on the complete dataset without the need for imputation or exclusion of cases due to missing values. SPSS26.0 (IBM, Armonk, New York, USA) was used to analyse the data. The continuous variables were tested for normal distribution using the Shapiro–Wilk test. Continuous data with a normal distribution were described as means ± standard deviations (SD). The independent sample *t*‐test (inter‐group comparison) and paired sample *t*‐test (intra‐group comparison) were implemented to analyse the differences. Continuous data with skewed distributions were presented as medians (interquartile range, IQR) and analysed using the Mann–Whitney *U* test. Categorical data were described as *n* (%) and analysed using the chi‐squared test (when the expected frequency was ≥ 5 in all cells of the contingency table), continuity correction chi‐squared test (when the expected frequency was < 5 in one cell of the contingency table) or Fisher's exact test (when the expected frequency was < 5 in more than one cell of the contingency table or the total sample size was < 40). Two‐sided *p* < 0.05 was considered statistically significant.

### 3.4 Ethical and Research Approvals

This study was approved by Ethics Committee of Scientific Research of Ruijin Hospital affiliated to Shanghai Jiao Tong University School of Medicine (approval No. 20240112091756999). The approval date is April 21, 2023. The patient's informed consent was waived due to the retrospective nature of this study.

## 4 Results

A total of 39 COVID‐19‐positive patients (16 females) were included, and 19 patients received the modified prone position. Sex, age, BMI, Charlson Comorbidity Index, APACHE II score at the beginning of the study, PaO_2_, PaCO_2_, SOFA score at the beginning of the study, time from onset of symptoms to admission, time from admission to the first prone position treatment, C‐reactive protein, D‐Dimer, lactic acid and mechanical ventilation were comparable between the two groups (all *p* > 0.05) (Table 1).

**TABLE 1:** Demographic characteristics.

Variables	Modified prone (*n* = 19)	Conventional prone (*n* = 20)	Test and statistic	Effect size	*p*
Sex			Fisher's exact	OR (95% CI)	0.333
Male	13 (56.5%)	10 (43.5%)		2.71 (0.60–7.81)	
Female	6 (37.5%)	10 (62.5%)		0.46 (0.13–1.67)	
Age (years)	75.11 ± 10.50 (55–89)	80.20 ± 8.31 (65–95)	*t* = −1.476	Cohen's *d* = 0.54	0.100
Complications			Fisher's exact	OR (95% CI)	1.000
No	2 (50.0%)	2 (50.0%)		1.00 (0.06–16.67)	
Yes	17 (48.6%)	18 (51.4%)		0.94 (0.29–3.05)	
BMI (kg/m^2^)	22.74 ± 3.34 (18.5–28.1)	22.56 ± 3.86 (17.9–29.3)	*t* = 0.153	Cohen's *d* = 0.05	0.880
Charlson comorbidity index	7.74 ± 2.08 (4–11)	7.85 ± 1.90 (5–10)	*t* = −0.178	Cohen's *d* = 0.06	0.860
APACHE II score	15.74 ± 6.62 (5–26)	17.05 ± 6.32 (7–28)	*t* = −0.634	Cohen's *d* = 0.20	0.530
Braden score	12.95 ± 2.39 (9–16)	12.80 ± 2.59 (8–15)	*t = 0.18*	Cohen's *d* = 0.06	0.860
PaO_2_ (kPa)	14.67 ± 4.15 (8.2–20.5)	13.18 ± 4.64 (6.5–21.0)	*t* = 1.059	Cohen's *d* = 0.34	0.297
PaCO_2_ (kPa)	4.57 ± 0.83 (3.1–6.0)	5.09 ± 1.22 (3.5–7.8)	*t* = −1.557	Cohen's *d* = 0.50	0.128
SOFA score	3.00 (2.00–4.00) [1–6]	3.00 (2.00–5.75) [1–8]	*U* = 152.5, *Z* = −0.740	*r* = 0.12	0.459
Symptom‐to‐admission time (days)	2.00 (1.00–5.00) [1–7]	3.00 (2.00–5.50) [1–10]	*U* = 130.0, *Z* = −1.621	*r* = 0.26	0.105
Admission‐to‐treatment time (hours)	18.00 (12.00–20.00) [10–24]	16.50 (10.00–23.00) [8–26]	*U* = 180.0, *Z* = −0.042	*r* = 0.01	0.966
C‐reactive protein (mg/L)	69.00 (41.05–154.00) [10–280]	119.00 (60.50–234.35) [25–350]	*U* = 130.0, *Z* = −1.433	*r* = 0.23	0.152
D‐dimer (mg/L)	1.66 (0.74–2.93) [0.3–5.1]	2.21 (1.32–4.10) [0.5–6.0]	*U* = 140.0, *Z* = −0.731	*r* = 0.12	0.465
Lactic acid (mmol/L)	1.59 (1.33–2.72) [0.8–3.5]	2.03 (1.39–2.82) [1.0–4.2]	*U* = 150.0, *Z* = −0.506	*r* = 0.08	0.613

Abbreviations: APACHE: Acute Physiology and Chronic Health Evaluation; BMI: body mass index; SOFA: sequential organ failure assessment.

The percutaneous oxygen saturations before and after treatment were comparable between the modified prone position group and the conventional prone position group (both *p* > 0.05). Transcutaneous blood oxygen saturation after both types of prone position was significantly increased compared to those before treatment (both *p* = 0.001) (Table 2).

**TABLE 2:** Transcutaneous blood oxygen saturation.

Group	Before treatment	After treatment	Test statistic (intra‐group)	Effect size (intra‐group)	Intra‐group *p*	Test statistic (inter‐group)	Effect size (inter‐group)	Inter‐group *p*
Modified prone (*n* = 19)	85.95 ± 4.53 (78–93)	95.11 ± 2.03 (91–98)	*t* = 9.21	Cohen's *d* = 2.54	0.001	*t* = 0.52	Cohen's *d* = 0.15	0.607
Conventional prone (*n* = 20)	86.65 ± 3.91 (79–94)	94.70 ± 2.32 (90–97)	*t* = 8.97	Cohen's *d* = 2.45	0.001	*t* = 0.58	Cohen's *d* = 0.17	0.565

The occurrence rate of pressure injury in the modified prone position group was significantly lower than that in the conventional prone position group (3 (25.0%) vs. 9 (75.0%), *p* = 0.044). The modified prone position group had two cases of pressure injury in the face and one case of pressure injury in the knee, while the conventional prone position group had five cases of pressure injury in the face and four cases of pressure injury in the knee. The eyelid oedema (2 (10.5%) vs. 9 (45.0%), *p* = 0.031) and facial oedema (5 (26.3%) vs. 13 (65%), *p* = 0.024) in patients with the modified prone position were significantly lower than in those with the conventional prone position. The occurrence site and stage of pressure injury, airway adverse events and brachial plexus injury were compared between the two groups (all *p* > 0.05) (Table 3). Daily continuous prone position time in patients with the modified prone position was significantly higher than in those with the conventional prone position (9.16 ± 3.01 vs. 6.50 ± 2.14, *p* = 0.003) (Table 4).

**TABLE 3:** Comparison of the incidence of airway adverse events, pressure injury, head oedema and brachial plexus injury between the two groups.

Variables		Modified prone position	Conventional prone position	Test statistic	Effect size	*p*
Pressure injury						
Rate of pressure injury	No	16 (59.3%)	11 (40.7%)	Fisher's exact	OR = 4.36 (1.05–18.10)	0.044
	Yes	3 (25.0%)	9 (75.0%)	Fisher's exact	OR = 0.23 (0.05–1.01)	0.044
Occurrence site	Face	2 (28.6%)	5 (71.4%)	Fisher's exact	OR = 1.00 (0.12–8.16)	1.000
	Knee	1 (20.0%)	4 (80.0%)	Fisher's exact	OR = 1.00 (0.08–12.50)	1.000
	Phase I	2 (28.6%)	5 (71.4%)	Fisher's exact	OR = 1.00 (0.12–8.16)	1.000
	Phase II	1 (20.0%)	4 (80.0%)	Fisher's exact	OR = 1.00 (0.08–12.50)	1.000
Stages	Phase III	0	0	—	—	
	Phase IV	0	0	—	—	
	Not installable	0	0	—	—	
	DTI	0	0	—	—	
Airway adverse events						
Artificial airway obstruction	No	6 (50.0%)	6 (50.0%)	Fisher's exact	OR = 1.00 (0.06–16.67)	1.000
	Yes	1 (50.0%)	1 (50.0%)	Fisher's exact	OR = 1.00 (0.06–16.67)	
Artificial airway dislodgement	No	7 (50.0%)	7 (50.0%)	Fisher's exact	N/A	1.000
	Yes	0	0	—	—	
Head oedema						
Eyelid oedema	Grade 0	17 (60.7%)	11 (39.3%)	Fisher's exact	OR = 6.95 (1.23–39.20)	0.031
	Grade 1	2 (18.2%)	6 (30.0%)			
	Grade 2	0	3 (15.0%)			
	Grade 3	0	0			
Facial oedema	Grade 0	14 (66.7%)	7 (33.3%)	χ^2^ = 9.40	Phi = 0.49	0.024
	Grade 1	5 (27.8%)	6 (30.0%)			
	Grade 2	0	5 (25.0%)			
	Grade 3	0	2 (10.0%)			
Incidence of brachial plexus injury	No	19 (48.7%)	20 (51.3%)	—	—	1.000
	Yes	0	0	—	—	

**TABLE 4:** Comparison of the duration of prone position treatment between the two groups.

Variables	Modified prone (*n* = 19)	Conventional prone (*n* = 20)	Test statistic	Effect size	*p*
Daily continuous prone position time (hours)	9.16 ± 3.01 (4–15)	6.50 ± 2.14 (3–12)	*t* = 3.19	Cohen's *d* = 1.02	0.003

A post hoc power analysis was conducted based on the observed incidence of pressure injury (15.8% in the modified group vs. 45.0% in the conventional group). Using Fisher's exact test with a two‐sided α = 0.05, the current sample size (*n* = 39) provided 62% power to detect this difference.

## 5 Discussion

This study suggests that compared to the conventional prone position, the modified prone position has significantly lower occurrence rates of pressure injury, eyelid oedema and facial oedema and significantly higher daily continuous prone position time. This result may provide a treatment option for COVID‐19 patients under mechanical ventilation in ICU.

The prone position promotes the patients' back alveolar recruitment, regulates the perfusion of the anterior chest wall and improves the ventilation‐blood flow ratio, thereby increasing the oxygenation index and the beneficial treatment effect [17]. Lucchini et al. found that the ‘dolphin’ prone position can be effectively applied to conscious patients with COVID‐19, which improved the tolerance to the treatment of conscious patients in the prone position [18]. The improved prone position suggested in the present study is based on a combination of the conventional prone position and the dolphin prone position. The head and foot of the bed were turned in opposite directions, positioning the patient's head where the legs were usually placed. As a result, the patient's chest could be elevated while the head could be kept in a relatively horizontal position.

The primary outcome is pressure injury. Many factors contribute to the occurrence of high‐pressure sores in patients with mechanical ventilation in ICUs, such as decreased sensory function, poor blood circulation, skin thinning and facial oedema. The focus of mechanical ventilation was usually on serious complications or stabilising vital signs [19, 20]. The occurrence of pressure injury was often ignored. However, hospital‐acquired pressure injuries are an important patient safety issue that cannot be overlooked [21]. Studies have shown that pressure injury leads to longer hospital length of stays and higher 30‐day readmissions [21, 22], causing negative sequelae for patients [23], nursing staff [24] and hospitals [25, 26]. Jang et al. found that COVID‐19‐positive patients in South Korea exhibited higher rates of pressure sores [27], suggesting the importance of pressure injury prevention in COVID‐19 patients. In the modified prone position, the head and tail of the bed are lifted to keep the patient's head at a relatively horizontal position, reducing the shear and friction forces caused by the lifting of the head of the bed. Foam accessories, such as pillows and other appliances, can effectively reduce the incidence of pressure sores and oedema on the patient's face, improve the quality of clinical care and ensure patient safety.

In the modified prone position, a U‐shaped pillow is utilised instead of the traditional cushion for the head. Due to the existence of a gap under the U‐shaped pillow, when the patient's face is turned downwards, there is still sufficient space to ensure the patient's breathing. Additionally, this position is convenient for the nursing staff to better observe the occurrence of pressure sores on the patient's face and eyelid oedema and timely intervene and adjust the position of the bed. The opening of the U‐shaped pillow and face are facing the same side, which can allow for the passage of catheters for oxygen therapy and respiratory support through the opening. This position can facilitate observation by the nursing staff and prevent the occurrence of catheter compression, distortion and accidental dislodgement during ventilation treatment for patients in prone positions.

The results showed that the tolerance to ventilation treatment in the modified prone position applied to patients with non‐invasive mechanical ventilation is significantly higher than that of the conventional prone position treatment. It is known to us that the human spine has four physiological curvatures ensuring the normal physiological function of the spine [28]. A previous study reported that when the patient lies in the prone position at the end of the bed, the elevation of the foot of the bed makes it more consistent with the physiological curvature of the human spine [18]. Thus, in this study, we applied this scientific view to the development of the proposed modified prone position. As mentioned, by raising the head and foot of the bed, the physiological curvature of the spine is placed in a better position. The patient's hands were placed on both sides of the patient's head and face, effectively improving the comfort of the patient and improving the tolerance to ventilation treatment.

## 6 Limitations

This study has several limitations. It is a retrospective single‐centre study, and the sample size was limited. The post hoc power analysis revealed a power of 62%. A total of 50 patients (25 per group) would be required to achieve 80% power. Future prospective studies with larger cohorts are needed to validate the findings. As a result, potential biases cannot be entirely excluded. Future investigations with an expanded sample size are essential to further investigate the efficacy of this modified prone position. Another limitation is that it is not possible to clearly distinguish whether the change in pressure injury incidence is due to a pillow or position. In subsequent studies, we plan to design a unique prone ventilation therapy nursing pad based on this research to confirm that such a position change is indeed effective.

## 7 Implications and Recommendations for Practice

The treatment of COVID‐19 patients with hypoxaemia in the prone position can effectively increase oxygenation and decrease intubation rate, thus improving prognosis and reducing mortality. However, patients in the prone position had poor tolerance to ventilation, resulting in a high incidence of unplanned extubation, pressure injuries and difficulty in nursing.

This study introduces a modified prone ventilation method, offering an effective and practical nursing solution for critical care nurses. In this modified prone position, the head is supported, and the bed's direction (head‐to‐foot alignment) is adjusted by reversing it, using a U‐shaped pillow during prone ventilation. This approach helps reduce pressure points on the face, facilitates catheter management through an opening in the pillow and aligns with spinal ergonomics to enhance patient comfort. The findings of this study provide a theoretical foundation for developing a nurse‐led prone management strategy aimed at optimising prone ventilation outcomes while minimising iatrogenic risks in the ICU setting.

## 8 Conclusion

Compared to the conventional prone position, the modified prone position may significantly reduce the occurrence of pressure injuries and improve patient tolerance in COVID‐19 patients under mechanical ventilation in the ICU. Moreover, the study suggested that it was simple and easy to operate with no need for additional tools or human resources, thus making it worthy of clinical promotion. However, multicentre clinical trials with large sample sizes are needed to validate the findings.

## Author Contributions

**Chen Huang:** conceptualisation, formal analysis, writing – original draft, writing – review and editing. **Wenwen Qi:** methodology and provided software. **Shuyuan Zhao:** data validation. **Xuelian Xu:** investigation. **Chaowei Yao:** resources. **Yirong Sun:** data curation. **Yan Jiang:** visualisation. **Xiaoye Wang:** photography. **Feng Jing:** supervision, project administration. **Enqiang Mao:** project administration. **Erzhen Chen:** funding acquisition. Wenwen Qi and Chen Huang made equal contributions, therefore Chen Huang is the first author of this article, and Wenwen Qi is the co‐first author of this article.

## Ethics Statement

This study was approved by the Ethics Committee of Scientific Research of Ruijin Hospital affiliated to Shanghai Jiao Tong University School of Medicine (approval no. 20240112091756999). The approval date is April 21, 2023. The patient's informed consent was waived due to the retrospective nature of this study.

## Consent

Since the patient consent statement is quite long, I have translated it into English and placed it at the end of this document. I certify that this research allows the reproduction of material from other sources.

## Conflicts of Interest

The authors declare no conflicts of interest.

## Data Availability

Data sharing is not applicable to this article as no new data were created or analyzed in this study.
